# Thermokinetic Study of Aluminum-Induced Crystallization of a-Si: The Effect of Al Layer Thickness

**DOI:** 10.3390/nano13222925

**Published:** 2023-11-10

**Authors:** Sergey M. Zharkov, Vladimir V. Yumashev, Evgeny T. Moiseenko, Roman R. Altunin, Leonid A. Solovyov, Mikhail N. Volochaev, Galina M. Zeer, Nataliya S. Nikolaeva, Oleg V. Belousov

**Affiliations:** 1 Kirensky Institute of Physics, Federal Research Center KSC SB RAS, Krasnoyarsk 660036, Russia; volochaev91@mail.ru; 2 Laboratory of Electron Microscopy, Siberian Federal University, Krasnoyarsk 660041, Russia; yumashev_vlad@mail.ru (V.V.Y.); e.t.moiseenko@ya.ru (E.T.M.); raltunin@gmail.com (R.R.A.); g-zeer@mail.ru (G.M.Z.); nn86@mail.ru (N.S.N.); ov_bel@icct.ru (O.V.B.); 3 Institute of Chemistry and Chemical Technology, Federal Research Center KSC SB RAS, Krasnoyarsk 660036, Russia; leosol@icct.ru

**Keywords:** amorphous silicon, Al/Si, nanolayer, multilayer film, metal-induced crystallization, aluminum-induced crystallization, kinetics, activation energy, enthalpy, simultaneous thermal analysis (STA)

## Abstract

The effect of the aluminum layer on the kinetics and mechanism of aluminum-induced crystallization (AIC) of amorphous silicon (a-Si) in (Al/a-Si)_n_ multilayered films was studied using a complex of in situ methods (simultaneous thermal analysis, transmission electron microscopy, electron diffraction, and four-point probe resistance measurement) and ex situ methods (X-ray diffraction and optical microscopy). An increase in the thickness of the aluminum layer from 10 to 80 nm was found to result in a decrease in the value of the apparent activation energy E_a_ of silicon crystallization from 137 to 117 kJ/mol (as estimated by the Kissinger method) as well as an increase in the crystallization heat from 12.3 to 16.0 kJ/(mol Si). The detailed kinetic analysis showed that the change in the thickness of an individual Al layer could lead to a qualitative change in the mechanism of aluminum-induced silicon crystallization: with the thickness of Al ≤ 20 nm. The process followed two parallel routes described by the n-th order reaction equation with autocatalysis (Cn-X) and the Avrami–Erofeev equation (An): with an increase in the thickness of Al ≥ 40 nm, the process occurred in two consecutive steps. The first one can be described by the n-th order reaction equation with autocatalysis (Cn-X), and the second one can be described by the n-th order reaction equation (Fn). The change in the mechanism of amorphous silicon crystallization was assumed to be due to the influence of the degree of Al defects at the initial state on the kinetics of the crystallization process.

## 1. Introduction

Metal-induced crystallization (MIC) is a promising method for obtaining high-quality polycrystalline silicon films from thin films of amorphous silicon (a-Si) at low temperatures, in particular, for the application in microelectronics (TFTs, technologies of optical information recording) [1,2,3]. Moreover, aluminum is an advantageous metal for the method of metal-induced crystallization. To illustrate, according to the phase diagram of the Al-Si system [4], aluminum, unlike copper [5], does not form compounds with silicon. In addition, silicon crystallization temperatures in Al/a-Si thin films are among the lowest ones (≥165 °C) [6,7,8]. Aluminum-induced crystallization (AIC) of Si is a prospective method of obtaining polycrystalline silicon for the application in low-cost photovoltaic devices [9,10,11].

Numerous studies are devoted to the mechanisms of AIC. In refs. [11,12,13,14], the AIC process in Al/a-Si bilayer thin films is shown to be accompanied by the aluminum-induced layer exchange, first detected and described in ref. [15]. Here, the crystallization process of amorphous silicon is due to the relaxation of elastic strain energy associated with the macrostress and microstrain caused by the layer exchange [1]. The crystallization of amorphous silicon in Al/a-Si thin films is highly dependent on the thickness of the aluminum layer [16,17]. For example, in ref. [18], it is shown that in Al/a-Si films with the silicon layer thickness of 100 nm, silicon crystallization upon annealing at 350 °C is observed only in the case of the aluminum layer thickness of >20 nm with the portion of crystallized silicon increasing with an increase in the aluminum thickness. In ref. [17], the authors show that upon silicon crystallization in Al/a-Si films with the silicon layer thickness of 160 nm, there exists the dependence of the morphology of crystalline silicon on the thickness ratio (t) of the silicon and aluminum layers: in the case of t_al_ ≥ t_a-Si_, crystalline silicon has better crystallinity, a smooth surface and preferred orientation. In refs. [19,20], the number of nucleation centers of crystalline silicon (c-Si) and orientation of crystallites c-Si were found to depend on the ratio of the Al and a-Si thicknesses upon aluminum-induced crystallization in Al/a-Si thin films. Moreover, it is proved that with the aluminum thickness <25 nm in Al/a-Si films, it is impossible to obtain a continuous c-Si film as a result of AIC [20]. Thus, a great number of studies are devoted to the influence of the initial layers of aluminum and amorphous silicon on the morphology of crystalline silicon formed as a result of AIC.

However, of interest is the influence of the aluminum thickness in Al/a-Si thin films on the kinetics of silicon crystallization in the process of AIC. There is a significant discrepancy in the literature data on the kinetics of aluminum-induced silicon crystallization in Al/a-Si thin films. To illustrate, in terms of silicon crystallization, numerous studies report varying estimates of the activation energy E_a_ (from 44.2 to 316.8 kJ/mol [13,16,21,22,23,24]). This wide range of the activation energy values of silicon crystallization presented by different authors is due to the fact that first of all, to estimate the E_a_ value, use was made of the data obtained by radically different methods, and the second reason is that the authors studied the samples obtained under different conditions and, thus, they had different morphology. In ref. [13], the estimates of the activation energy (172.8 kJ/mol) of polycrystalline silicon nucleation during AIC in Al/Si films (with the Al thickness of 360 nm and Si thickness of 410 nm) were obtained as a result of processing optical observations. Similar values (169.9 kJ/mol) were obtained in ref. [16] based on the X-ray diffraction data (XRD) for Al/Si films (the Al thickness being 50 nm and Si thickness of 200 nm). In ref. [22], based on the XRD data, the activation energy of silicon crystallization in Al/Si films (with the Al thickness being 30 nm and that of Si being 200 nm) was estimated to be 134.4 kJ/mol. Most data were obtained by the following methods: XRD, optical observations, etc. However, the most reliable method to obtain the kinetic parameters such as activation energy is the differential scanning calorimetry (DSC). There are only a few studies where the kinetics of silicon crystallization was investigated by this method. The authors in refs. [23,24] present the investigation results for AIC in (Al/Si)_n_ multilayered thin films with the thickness of an individual Al layer being 3–6 nm and that of silicon being 6–20 nm. This resulted in obtaining the enthalpy values (12 kJ/mol) and activation energy (115.2 kJ/mol) of aluminum-induced crystallization.

The reliable physicochemical information allows developing approaches of targeted synthesis of functional materials. Thus, the present study is aimed at investigating the influence of the thickness of an individual aluminum layer in (Al/a-Si)_n_ multilayered films on the kinetics and mechanism of aluminum-induced crystallization of amorphous silicon by means of the DSC method.

## 2. Materials and Methods

### 2.1. Sample Preparation

To study the process of Al-induced crystallization of Si by the method of simultaneous thermal analysis (STA), a series of (Al/a-Si)_n_ multilayered films was obtained (with the number of bilayers being n = 35 ÷ 60) with different thicknesses of the aluminum layers equal to 10, 20, 40, 80 nm; and a fixed thickness of the amorphous silicon layer equal to 80 nm. The number of layers (n) was chosen so as to provide the sufficient sample weight for STA (10–20 mg for one measurement). Also, in order to carry out particular investigations by the methods of electron and optical microscopy and to measure electrical resistivity, bilayer films were obtained (i.e., n = 1) with the same thicknesses of the aluminum and silicon layers. The (Al/a-Si)_n_ films were obtained by magnetron sputtering in high vacuum in the pulse DC mode in the case of aluminum sputtering and in the DC mode in the case of silicon. The first layer to be deposited was aluminum, which was followed by silicon. The basic residual pressure was 3.7 × 10^−4^ Pa and the argon pressure during sputtering was 0.44 Pa. The high-purity materials (Girmet Ltd., Moscow, Russian Federation) Al (99.997 wt.%) and monocrystalline Si (100) n-type (silicon 99.999 wt.%) were used as targets. The film thickness was controlled using an INFICON SQC-310 thin film deposition controller. The sputtering rate for the aluminum layers was 0.18 nm/s, and that for silicon was equal to 0.17 nm/s. The glass substrates covered by a thin layer of Sigma-Aldrich 430102 Polystyrene (Sigma-Aldrich, Burlington, VT, USA) were used for the (Al/Si)_n_ multilayered film deposition. After the deposition, the obtained multilayered films were separated from the substrate by dissolving the polymer layer in chemically pure butyl acetate (≥99.5%) followed by rinsing in chemically pure acetone (≥99.8%), and vacuum drying at 3.7 × 10^−4^ Pa.

### 2.2. Electron Microscopy

The microstructure and local elemental composition of the samples were studied using a JEM-2100 (JEOL Ltd., Tokyo, Japan) transmission electron microscope equipped with an Oxford Inca x-sight energy-dispersive spectrometer (EDS) at an accelerating voltage of 200 kV. The elemental composition of the multilayered films was studied using a JSM-7001F (JEOL Ltd., Tokyo, Japan) scanning electron microscope equipped with an Oxford Inca Energy 350 at an accelerating voltage of 15 kV.

The investigations of the microstructure, phase and elemental composition of the (Al/Si)_n_ multilayered films were carried out using the methods of transmission electron microscopy (TEM), selected area electron diffraction (SAED), and energy-dispersive spectroscopy (EDS) using the transmission electron microscope JEOL JEM-2100. The electron diffraction patterns were interpreted using the software Gatan Digital Micrograph v. 1.71.38, CrysTBox [25,26] and databases ICDD PDF 4+ [27] and Pearson’s Crystal Data [28].

Cross-sections of the samples for transmission electron microscopy were prepared by a focused ion beam (FIB) using a Hitachi FB-2100 (Hitachi High-Tech, Tokyo, Japan) (40 kV accelerating voltage) with the subsequent Ar+ polishing at 0.5 kV.

To investigate the process of phase formation during the solid-state reaction, the (Al/Si)_n_ films were heated using a JEOL JEM-2100 heating sample holder. Simultaneously with heating, SAED patterns were registered, with the temperature of the sample being measured. The method was successfully used to investigate the phase formation during the solid-state reaction in different thin-film nanosystems: Al/Cu [29,30], Al/Pt [31], Al/Ag [32], Cu/Au [33], Al/Fe [34], Cu/Si [35], Fe/Si [36], Fe/Pd [33,37,38], and Co-ZrO_2_ [39].

### 2.3. X-ray Diffraction

The powder X-ray diffraction (XRD) data were collected at 25 °C on a PANalytical X’Pert PRO diffractometer (PANalytical BV, Almelo, The Netherlands) operating with CuK_a_ radiation (1.541874 Å) in the scan range from 10° to 140° 2*θ* (step width = 0.02° 2*θ*; time per step 10 s). The phase composition of the films and microstructural characteristics of individual phases were determined using the quantitative X-ray powder diffraction analysis with the full-profile Rietveld method [40] and derivative difference minimization [41].

### 2.4. Resistivity Measurements

The samples were heated from room temperature to 300 °C under the conditions of high vacuum (3.7 × 10^−4^ Pa). The sample temperature was controlled with a chromel–alumel thermocouple. The electrical resistivity and temperature were simultaneously measured with a Keithley 2450 SourceMeter (Tektronix, Beaverton, OR, USA) and a Keithley DMM6500 digital multimeter (Tektronix, Beaverton, OR, USA). To measure the resistivity, use was made of the four-point probe method with the four probes aligned on one side of the film sample located on a glass substrate.

### 2.5. Simultaneous Thermal Analysis

Simultaneous thermal analysis (STA) of the multilayer film samples in the Al-Si system was performed with a thermal analyzer Jupiter STA 449C (NETZSCH Gruppe, Selb, Germany) in Pt-Rh crucibles with perforated lids. Simultaneous recording of the change in the weight (by thermogravimetry (TG)) and in the heat flow (by differential scanning calorimetry (DSC)) was made in the range of 40–300 °C at different heating rates: 2.5, 5, 7.5 and 10 °C/min in the dynamic argon atmosphere (grade 99.999%) with the total flow of 50 sccm. The thin film samples were separated from the substrate and placed into a crucible, the sample weight for one experiment being 12 ± 1 mg. For processing the primary thermoanalytical data, use was made of the software NETZSCH Proteus v. 4.8.4. The kinetic parameters of the phase formation were simulated and estimated using the software package NETZSCH Thermokinetics 3 v. 2006.08. The heat flow calibration of the DSC-TG sensor was performed by the method (DIN 51007:1994-06 Thermal analysis; differential thermal analysis; principles) with the relative error not exceeding 2%.

## 3. Results

### 3.1. TEM and XRD

**Figure 1:**
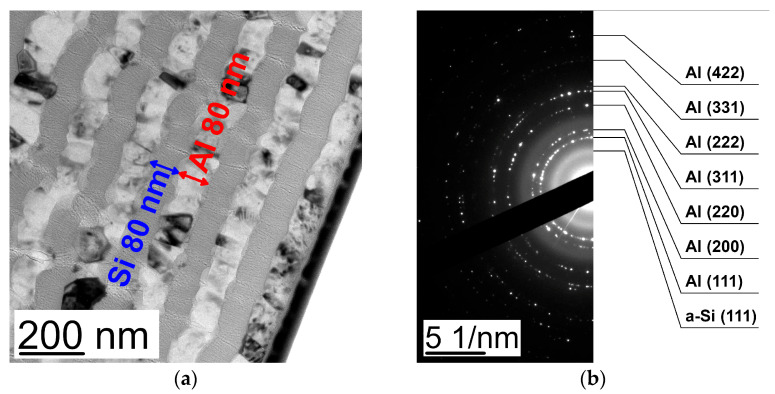
The cross-section TEM image (**a**) and ED pattern (**b**) obtained from the (Al 80 nm/Si 80 nm)_n_ multilayered thin film at the initial state.

The analysis of the cross-section TEM images of the (Al/a-Si)_n_ multilayered films with the thickness of the Al layer being 80 nm and that of the Si layer equal to Si 80 nm (Figure 1a) in the initial state showed the size of the aluminum crystallites to be 60–70 nm. The analysis of the electron diffraction patterns (Figure 1b) demonstrated the full range of polycrystalline reflections characteristic for fcc Al (PDF 4+ card #00-004-0787, space group Fm-3m, a = 4.0494 Å) as well as an amorphous halo corresponding to the a-Si phase (PDF 4+ card #00-005-0565, space group Fd-3m, a = 5.4301 Å).

The elemental analysis of the (Al/a-Si)_n_ multilayered thin films with different thicknesses of the aluminum layers and a fixed thickness of the silicon layer was performed by EDS. As a result, the aluminum and silicon content was determined in the (Al/a-Si)_n_ films with the aluminum thicknesses equal to 10, 20, 40 and 80 nm and the silicon thickness equal to 80 nm (Table 1).

**Figure 2:**
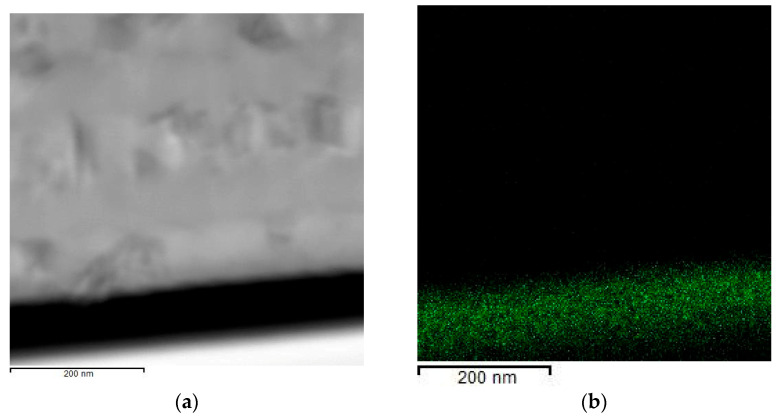

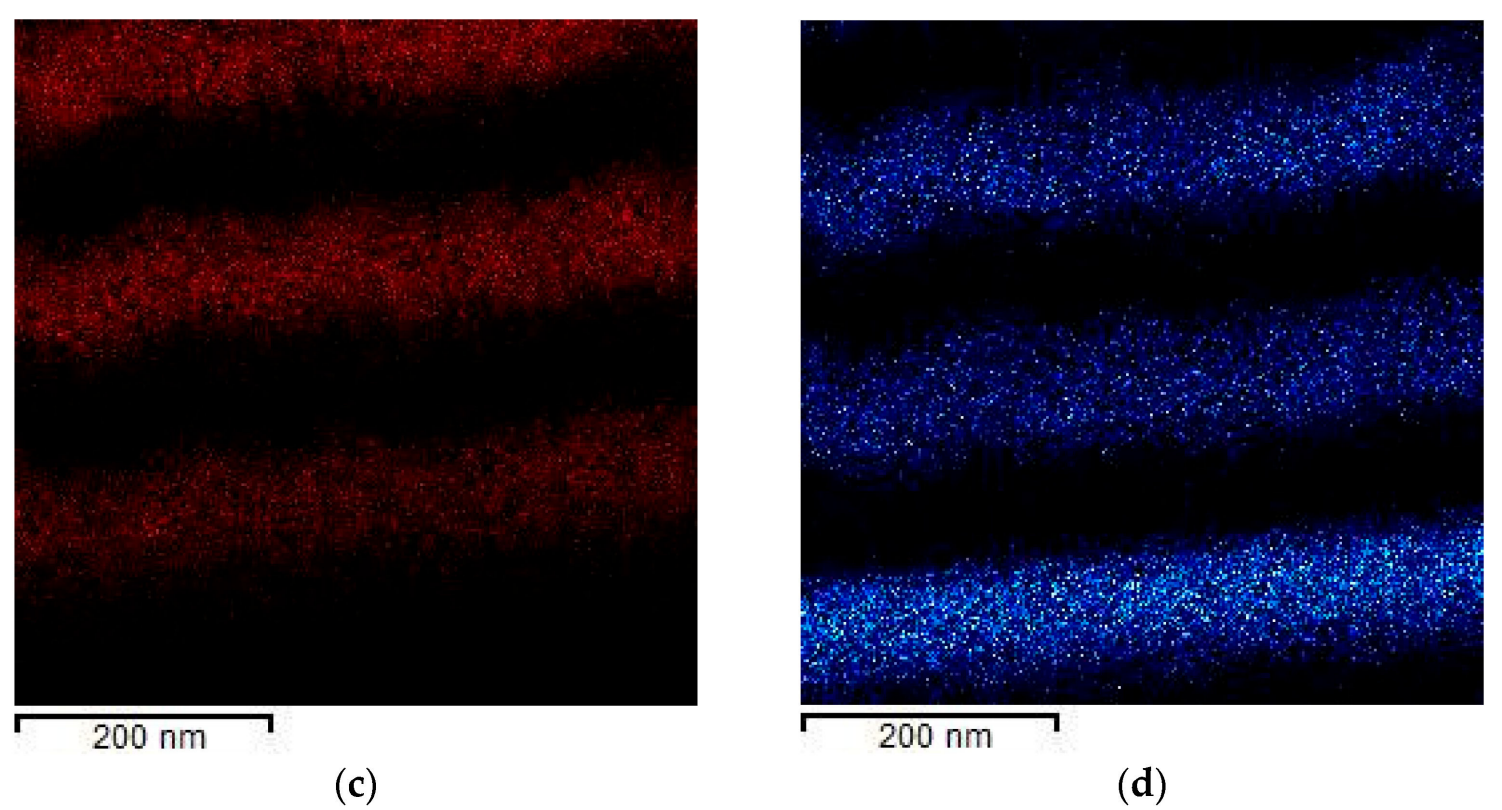
The STEM image (**a**) and EDS mapping ((**b**)—W, (**c**)—Al, (**d**)—Si) obtained from the (Al 80 nm/Si 80 nm)_n_ multilayered film at the initial state.

Figure 2a shows the cross-section STEM image of the (Al/a-Si)_n_ film with the aluminum layer thickness equal to 80 nm, and Figure 2b–d presents EDS mapping for W, Al and Si, respectively. Tungsten, which is present in the element-distribution maps, is a protective layer applied during the sample preparation procedure.

**Figure 3:**
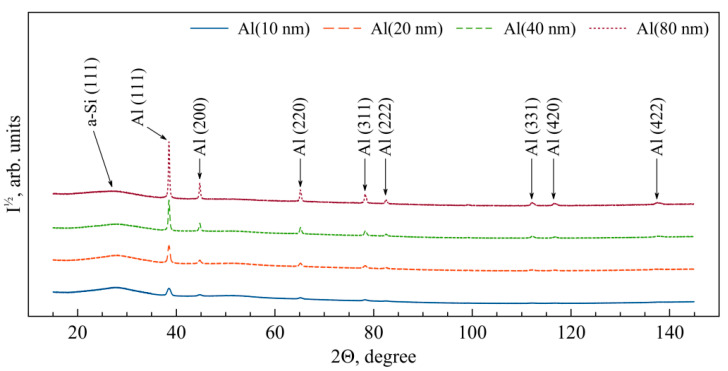
The XRD patterns obtained from the (Al/a-Si)_n_ multilayered films with the Al layer thickness 10–80 nm at the initial state.

The analysis of the X-ray diffraction patterns obtained from the (Al/a-Si)_n_ multilayered thin films with different Al thicknesses at the initial state (Figure 3) showed the observed diffraction reflections to correspond to the fcc phases of aluminum and amorphous silicon (a-Si). Here, the following preferred orientation was observed: the Al (111) planes were parallel to the plane of the substrate. As a result of processing the X-ray diffraction patterns, the following was estimated: Al lattice constants (*a*), average size of the crystallites (❬*D*❭), size distribution widths of the crystallites (δ❬*D*❭), as well as microstrain (❬*ε_Al_*❭_111_) in the (Al/a-Si)_n_ multilayered films (Table 2). With a decrease in the aluminum layer thickness in the multilayer films, the values of microstrain in the aluminum layer (❬*ε_Al_*❭_111_) were observed to increase. Based on the obtained values of the aluminum lattice constant, the values of microstrains (σ_Al_) were calculated in the aluminum layer (see Table 2). It is worth noting that there are no significant macrostresses (σ_Al_) within the aluminum layer in the (Al/a-Si)_n_ films at the initial state. The average size of the aluminum crystallites obtained for the film with the aluminum thickness of 80 nm was equal to 61.3 nm, which is in good agreement with the TEM data (Figure 1a).

**Figure 4:**
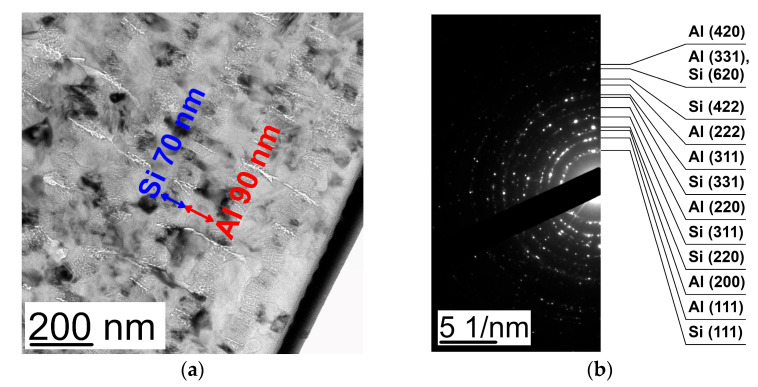
The cross-section TEM image (**a**) and ED pattern (**b**) obtained from the (Al 80 nm/Si 80 nm)_n_ multilayered thin film after heating up to 300 °C.

The analysis of the TEM images (Figure 4a) and electron diffraction patterns (Figure 4b) obtained from the cross-sections of the (Al/Si)_n_ multilayered films with the thicknesses of both Al and Si layers equal to 80 nm (Figure 1a) after heating to 300 °C showed the films to consist of crystallites of fcc aluminum and crystalline silicon (c-Si) with the size equal to 60–80 nm with the layered structure of the sample being retained.

**Figure 5:**
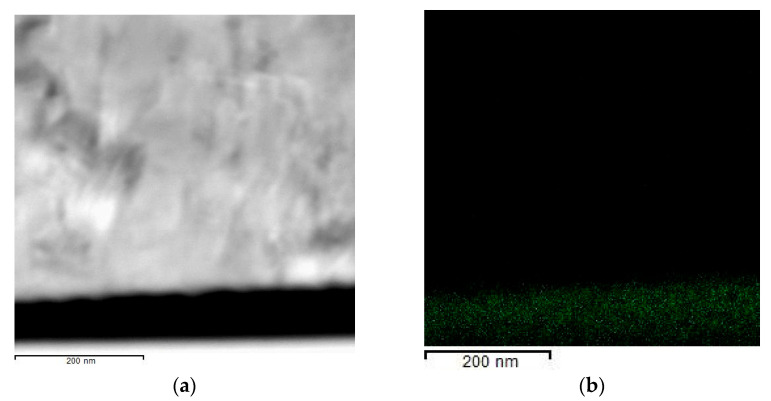

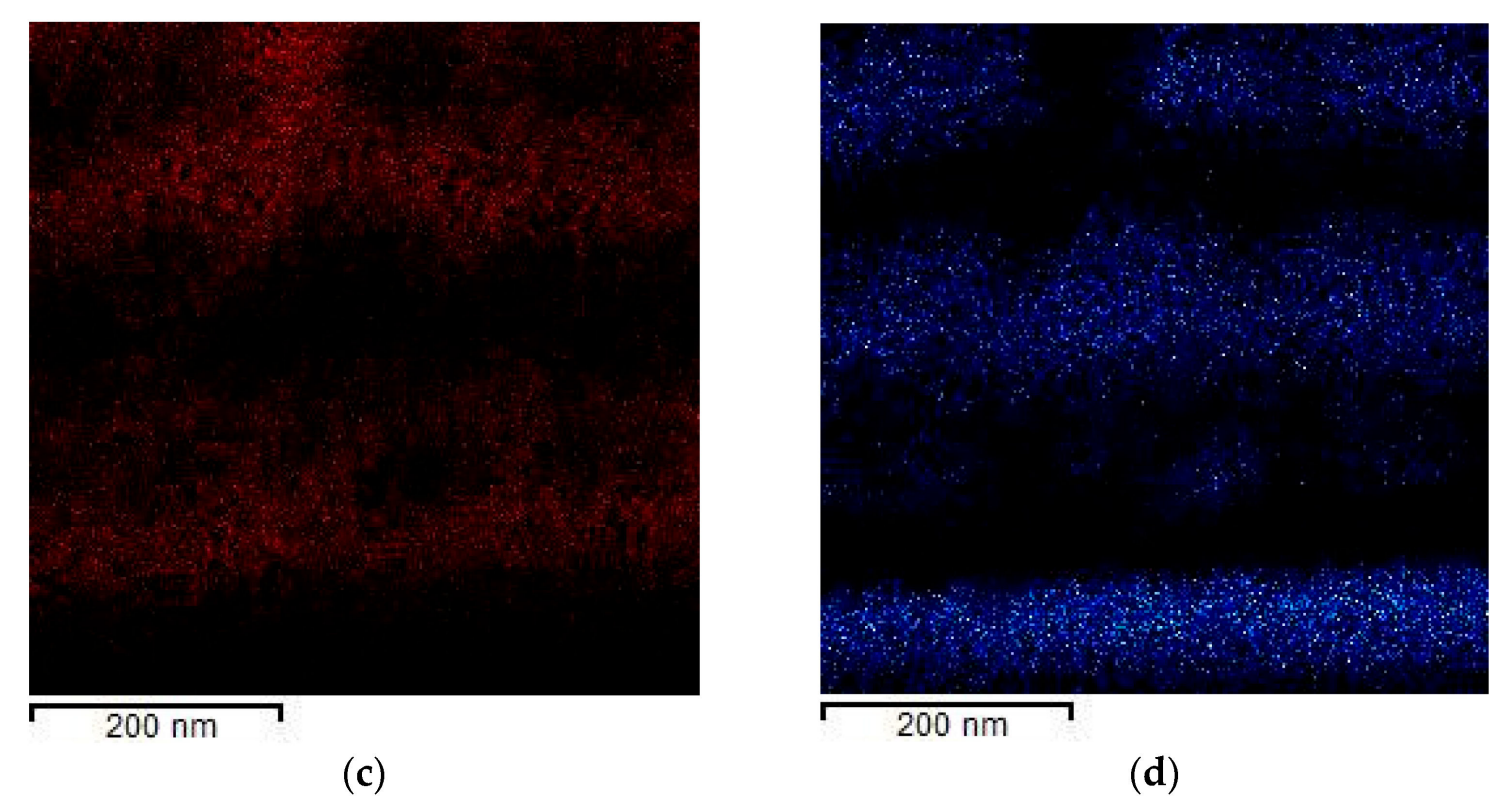
The STEM image (**a**) and EDS mapping ((**b**)—W, (**c**)—Al, (**d**)—Si) obtained from the (Al 80 nm/Si 80 nm)_n_ multilayered film after heating up to 300 °C.

Figure 5a presents the STEM image of a cross-section of the (Al/Si)_n_ film with the aluminum layer thickness equal to 80 nm after heating to 300 °C, and Figure 5b–d shows the distribution maps of W, Al and Si, respectively.

**Figure 6:**
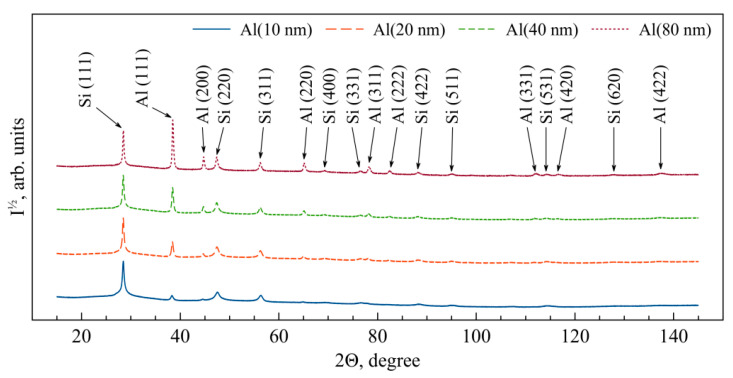
The XRD patterns obtained from the (Al/Si)_n_ multilayered films with the Al layer thickness of 10–80 nm after heating up to 300 °C.

The analysis of the X-ray diffraction patterns obtained from the (Al/Si)_n_ films with different thicknesses of the aluminum layer after heating to 300 °C (Figure 6) showed the observed diffraction reflections to correspond to the phases of fcc Al and c-Si. In this case, there exists the preferred orientation: the Al (111) and Si (111) planes are parallel to the plane of the substrate. Processing the X-ray diffraction patterns allowed estimating the lattice constants of Al and c-Si, and the crystal lattice of silicon (c-Si) was found to have rhombohedral distortions (see Table 3).

With the decrease in the aluminum layer, the presence of defects in c-Si obtained by AIC was observed to increase, and one could also observe a decrease in the lattice constant and an increase in the distortion degree of the c-Si lattice. Moreover, with the decrease in the aluminum layer in the films, the macrostresses increased: tensile stresses in aluminum and compressive ones in silicon (see Table 3). The values of macrostresses (σ) were calculated by Hooke’s law using the obtained values of the crystal lattice constants of aluminum and silicon as well as the values of the Young’s modulus typical for polycrystalline aluminum and silicon (see Table 3). The obtained values of the macrostresses in the silicon and aluminum layers in terms of their order of magnitude are characteristic for heteroepitaxy in thin film systems [43], and they were observed in Al/Si thin films earlier [44,45]. It is worth noting that it was impossible to estimate microstrain in the (Al/Si)_n_ films after heating due to a high degree of defects. As a consequence, it was possible to observe a complex, anisotropic-asymmetric broadening of the diffraction peaks. At the qualitative level, it is possible to conclude that the degree of defects decreases with an increase in the aluminum layer thickness.

### 3.2. Electrical Resistivity

**Figure 7:**
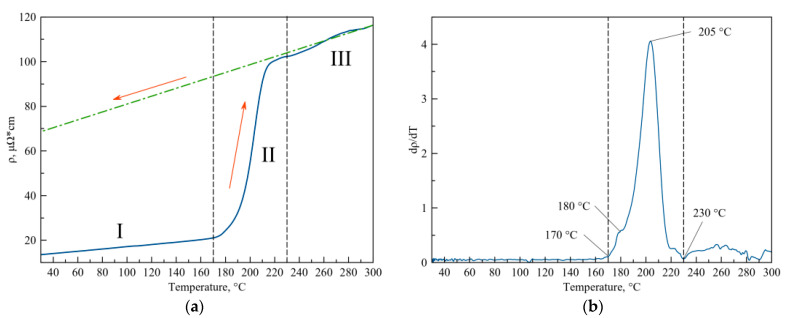
The change in the resistivity value upon heating the Al sample (80 nm)/a-Si (80 nm) at a heating rate of 5 °C/min (**a**) and temperature dependence of d ρ/dT (**b**).

The analysis of the behavior of the resistivity value (ρ) upon heating the Al sample (80 nm)/a-Si (80 nm) at a rate of 5 °C/min (see Figure 7a) shows that the change in the ρ value during heating can be divided into three steps: (I) linear dependence in the range from room temperature to 170 °C; (II) dependence of the type of the S-shaped curve in the range from 170 to 230 °C; (III) dependence close to the linear one in the range from 230 to 300 °C. The change in the ρ value during the sample cooling from 300 °C down to room temperature is linear, which is shown by the dash–dotted line in Figure 7a. Figure 7b presents the temperature dependence of d ρ/dT calculated based on the experimental data for ρ(T), which is given in Figure 7a.

The ρ value of the Al sample (80 nm)/a-Si (80 nm) in the initial state at room temperature is equal to 13.2 μΩ·cm, and it increases linearly in the process of heating at 170 °C ρ = 21.1 μΩ·cm (see Figure 7a, step I). Furthermore, during the sample heating (see Figure 7a, step II), the change in the ρ value is no longer linear, and it starts sharply increasing to the value of 102 μΩ·cm at 230 °C. Heating in the range from 230 to 300 °C (see Figure 7a, step III) is accompanied by a change in the ρ value, which is close to the linear one, and at 300 °C, it reaches the value 116.4 μΩ·cm. In the process of sample cooling, the ρ value changes linearly, and at room temperature, it is equal to 67.8 μΩ·cm.

### 3.3. Optical Microscopy

**Figure 8:**
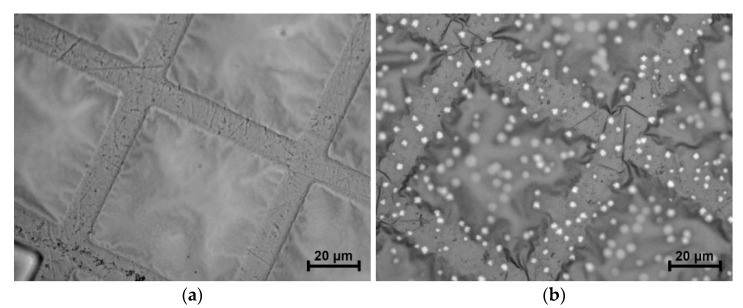
The reflected optical microscopy images of Al (80 nm)/a-Si (80 nm) at the initial state (**a**) and after heating up to 170 °C (**b**).

Figure 8a,b shows the reflected optical microscopy images of the bilayer film of Al (80 nm)/a-Si (80 nm). The film is located on an electron microscopy support grid. At the initial state (see Figure 8a), the film has no vivid structural inhomogeneities (the contrast observed in Figure 8a is due to the film warping). The image of the Al/Si film after heating to 170 °C is presented in Figure 8b, where the bright light spots ~1 μm in size correspond to the areas of crystallized silicon.

### 3.4. Simultaneous Thermal Analysis

**Figure 9:**
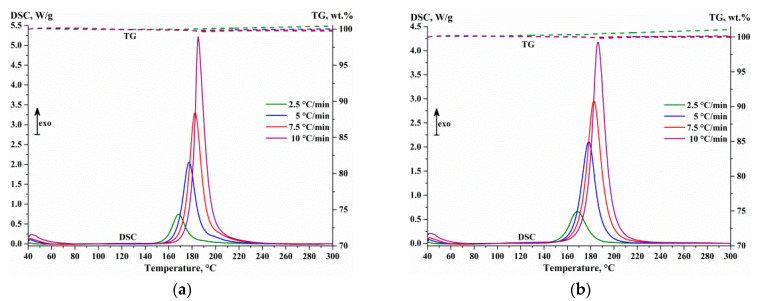

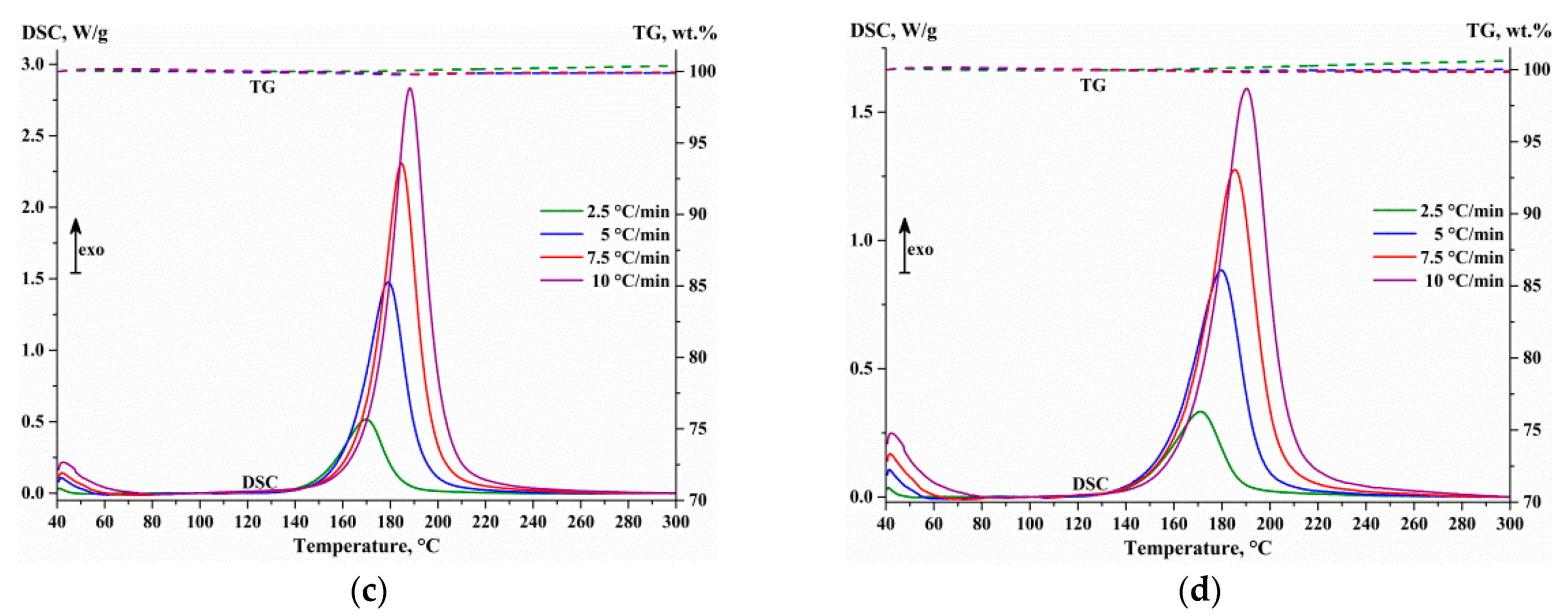
The DSC-TG curves obtained from the (Al/a-Si)_n_ multilayered films with the aluminum thickness of 10 (**a**), 20 (**b**), 40 (**c**) and 80 nm (**d**) upon heating up to 300 °C.

Simultaneous thermal analysis of the (Al/a-Si)_n_ multilayer thin film samples with different thicknesses of the individual aluminum layer (10, 20, 40 and 80 nm) with the fixed thickness of the individual silicon layer Si (80 nm) showed (Figure 9) the presence of a monomodal exothermal peak on the DSC curve in the temperature range of 80–300 °C. This peak corresponds to the process of amorphous silicon crystallization [15]. Here, any noticeable changes in the sample weight are almost absent on the thermogravimetric curves (Figure 9).

The heat flow measured by the DSC method in the non-isothermal mode is, in the course of the reaction, proportional to the rate of the solid-state transformation:

$$\frac{d\alpha }{\mathrm{dt}}=\frac{1}{Q_{0}}\frac{\mathrm{dQ}}{\mathrm{dt}}$$

where α is the rate of conversion (0 ≤ α ≤ 1);

t is the time;

Q_0_ is the total amount of heat released/absorbed during the reaction;

dQ/dt is the heat flow.

In the present study, the rate of conversion α corresponds to the portion of amorphous silicon transformed into crystalline silicon. The rate of conversion α was calculated as a portion of heat per weight unit of the sample released at the time moment t during the solid-state reaction [46]:

$$\alpha \left(t\right)=\frac{\int_{t_{S}}^{t}\left[\mathrm{DSC}\left(t\right)-\mathrm{Baseline}\left(t\right)\right]\;\mathrm{dt}}{\int_{t_{S}}^{t_{F}}\left[\mathrm{DSC}\left(t\right)-\mathrm{Baseline}\left(t\right)\right]\mathrm{dt}}$$

where t is the actual time;

t_S_ is the starting time;

t_F_ is the time at the end of the reaction;

DSC(t) is the differential scanning calorimetry signal;

Baseline(t) is the baseline signal belonging to the reaction peak.

For small weighed portions of the material of about 10–15 mg and lower and heating rates lower than 20 °C/min, this dependence is close to the linear one [47]. This allows neglecting the distortions of the heat flow (those of the peak shape and location of the maximum) arising due to the contribution of the heat capacity and thermal inertia of the sample material, crucible and DSC-TG sensor.

The model-free methods of thermal kinetics allow one to preliminarily estimate the kinetic parameters E_a_ and log(A) by the Kissinger method [48]. Using the Friedman method [49], it is possible to study the complexities of the observed process, namely the presence of more than one step, the existence of diffusion control, etc., taking into account the change in E_a_ and log(A), depending on the rate of conversion.

Table 4 shows the characteristic temperatures of the DSC peak of a-Si crystallization in the thin films samples of the (Al/a-Si)_n_ system: T_conversion 1%_ is the temperature at the rate of conversion α = 0.01; T_onset_ is the point of the baseline intersection to the left from the peak with the tangent in the inflection point of the leading edge of the DSC peak; T_max_ corresponds to the maximum reaction rate; T_end_ is the temperature of termination of the solid-state transformation. The process of crystallization of amorphous silicon is found to occur in all the (Al/a-Si)_n_ multilayered films under study, which is confirmed by the DSC data analysis (see Figure 9) revealing the absence of additional heat effects at temperatures higher than T_end_ as well as by the XRD data analysis (see Figure 6). Upon increasing the sample heating rate (Table 4) from 2.5 to 10 °C/min, the onset temperature T_onset_ of the solid-state interaction in the (Al/a-Si)_n_ sample is shifted to the range of high temperatures (Table 4): T_conversion 1%_ from 133–152 to 147–170 °C; T_onset_ from 145–157 to 168–179 °C; and the characteristic temperature T_max_ behaves in the same manner: it is shifted from 169–171 to 185–190 °C. The temperature T_end_ is observed in the range of 180–208 °C.

Apart from the effect of the heating rate on the location of the characteristic temperatures of the DSC peak, the thickness of the aluminum layer in the (Al/a-Si)_n_ multilayer system is of significance. As shown in Table 4, with the increase in the aluminum layer thickness from 10 to 80 nm, the onset temperature of a-Si crystallization (T_onset_) is monotonously shifted to the range of low temperatures by approximately 20 °C independent of the heating rate. However, the temperatures of the maximum reaction rate T_max_ and those of the reaction termination are, on the contrary, shifted to the range of high temperatures by approximately 2–5 °C and 7–12 °C, respectively. The shape of the peak on the DSC curves changes with the change in the aluminum layer thickness (Figure 9a–d): the leading edge of the peak becomes flatter, and, as a consequence, its asymmetry increases. The increased asymmetry of the DSC peak can be due to the appearance (or temperature shift) of an additional peak component.

The process of silicon crystallization in the (Al/a-Si)_n_ multilayered films was accompanied by the exothermic effect. The enthalpy value (see Table 4), calculated taking into account the mass fraction of silicon in the sample according to the EDS data (see Table 1), amounted to: −ΔH = 12.3–16.0 kJ/(mol Si). The obtained enthalpy values for the samples with the individual aluminum layer thickness of 10–20 nm (−ΔH = 12.3–13.3 kJ/mol) are in good agreement with the literature data for the process of amorphous silicon crystallization (−ΔH = 11.3–13.4 kJ/mol [24,50,51]), while for the samples with the aluminum thickness of 40–80 nm, significantly higher enthalpy values of silicon crystallization were observed (−ΔH = 15.8–16.0 kJ/mol).

## 4. Discussion

### 4.1. Resistivity

The ρ value of the thin-film sample of Al (80 nm)/a-Si (80 nm) at the initial state is 13.2 μΩ*cm (see Figure 7a), which corresponds to the electrical resistivity of the aluminum films, which is about 80 nm in thickness with the crystallite size ≈20–50 nm [52]. The calculation of the temperature coefficient of electrical resistivity (TCR) α, corresponding to the linear change in the ρ value at the initial stage of sample heating in the range from T_room_ to 170 °C (see Figure 7a, step I), shows that α = 0.0041 °C^−1^. The obtained value of α is well correlated with the value characteristic of pure aluminum, α_Al_ = 0.0039–0.0046 °C^−1^ [53,54]. This information allows us to conclude that in the case of measuring the electrical resistivity of the Al/Si sample using the four-point probe method, the measured ρ value is primarily determined by the electrical conductivity of the aluminum layer.

The sharp increase in the ρ value within the temperature range of 170–230 °C (see Figure 7a, the “S”-shaped increase in the ρ value at step II) is accounted for by silicon diffusion into the aluminum layer as well as by the crystallization of amorphous silicon. It is worth noting that both the onset of the change in the ρ value at T = 170 °C (which corresponds to the rate of conversion α = 0.28, estimated based on the DSC curve, see Figure 9d) and the maximum growth rate of ρ at T = 205 °C (rate of conversion α = 0.95) as observed on the d ρ/dT curve (see Figure 7b) are shifted toward higher temperatures as compared with the DSC curve obtained at the same heating rate (see Figure 9d, the heating rate of 5 °C/min). This fact indicates that silicon at the initial stage diffuses into the aluminum layer only in certain areas rather than uniformly over the entire area of the silicon–aluminum interface, which is confirmed by the optical microscopy data (see Figure 8b), showing the silicon crystallization centers to be far apart from each other. As a result, at the initial stage of diffusion and crystallization of silicon, this does not affect the ρ value at all, since it does not lead to the disruption of communication channels between the aluminum crystallites. It is only when the process of diffusion and crystallization of silicon reaches its maximum T = 180 °C (see the DSC curve in Figure 9d, the heating rate of 5 °C/min, the rate of conversion α = 0.58) that a significant increase in the ρ value begins (see Figure 7a,b). This means that the electrical contact between individual aluminum grains deteriorates due to silicon being accumulated at the boundaries of a significant number of aluminum grains, which leads to the deterioration in electrical conductivity. The “S”-shaped growth of the ρ value reaches its maximum at T = 205 °C and almost finishes at 230 °C (see Figure 7a,b), which coincides with the temperature at which the heat release process ends on the DSC curve (see Figure 9d).

It can be noted that the ρ value in the range from 230 to 300 °C (see Figure 7a, step III) almost coincides with the curve corresponding to sample cooling (see Figure 7a). This allows one to conclude that by the end of step II, a sample is formed whose structure does not undergo any significant changes upon further heating up to 300 °C. The TCR value, calculated based on the linear change in ρ upon cooling the sample from 300 °C to room temperature, amounts to 0.00261 °C^−1^.

Thus, based on the analysis of data on the behavior of the electrical resistivity ρ during heating of the thin-film Al/Si sample, one can conclude that heating the sample to 300 °C resulted in its morphology having the following form: an aluminum layer consisting of aluminum particles surrounded a thin layer of silicon and a layer of crystalline silicon. It should be noted that similarly to the initial state, the establishment of the resistivity value is determined primarily by the conductivity of the aluminum layer.

### 4.2. Estimation of the Kinetic Parameters Using Non-Isothermal Model-Free Methods

#### 4.2.1. Kissinger Analysis

The model-free Kissinger–Akahira–Sunose method [55] allows one to preliminarily estimate E_a_ and log(A) using the temperature values T_m_ at the maximum rate of the observed process with different rates of linear heating β = dT/dt = const. In our study, it is sufficient to use the special case of the Kissinger–Akahira–Sunose method—the classical Kissinger method [48]:

$$\ln\left(\frac{\beta_{i}}{T_{m,i}^{2}}\right)=\;\ln\left(-\frac{\mathrm{AR}}{E_{a}}f\prime \left(\alpha_{m}\right)\right)-\frac{E_{a}}{{\mathrm{RT}}_{m,i}},$$

where T_m,i_ is the temperature corresponding to the rate of conversion α_m_ at the reaction peak maximum for the i-th measurement for the classical Kissinger method;

β_i_ = dT/dt is the linear heating rate for the i-th measurement;

E_a_ is the apparent activation energy;

A is the apparent pre-exponential factor in the Arrhenius equation;

R is the universal gas constant;

f’(α) = df(α)/dα, where f(α) is the reaction type according to refs. [56,57]; for the reaction of the 1st order f(α) = (1 − α), f’(α) = −1.

**Figure 10:**
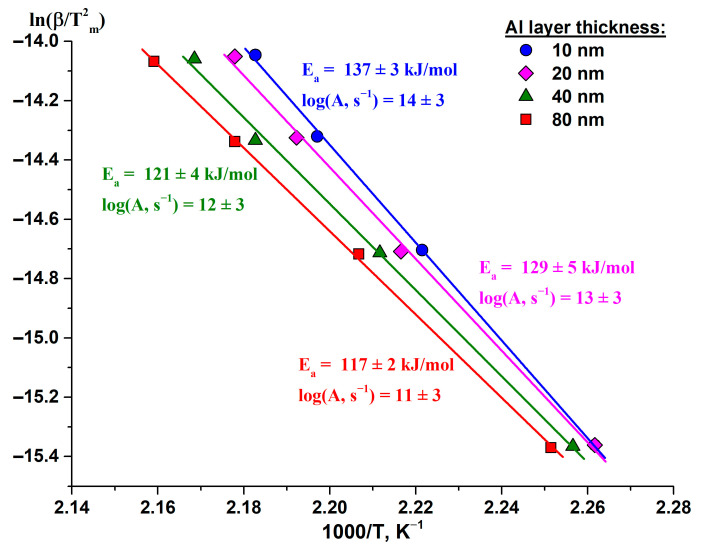
The Kissinger plots for the DSC peaks obtained from the (Al/Si)_n_ multilayered films with the aluminum thickness of 10–80 nm upon heating up to 300 °C.

To estimate the kinetic parameters in the (Al/a-Si)_n_ thin films, we plotted the ln(β/$T_{m}^{2}$) dependences on 1/T_m_ (Figure 10). The tangent of the tilt angle in the Kissinger dependences, as well as in the case of the Arrhenius dependence, is determined by the activation energy and corresponds to the E_a_/R value. The common logarithm of the pre-exponential factor A was calculated from the value corresponding to the intersection with the Y axis (Intercept): log(A) = [Intercept + ln(E_a_/R)]/ln10.

The initial estimation of the kinetic parameters (apparent activation energy and pre-exponential factor) using the Kissinger method [48] up to the temperatures of the DSC peak maximum (Figure 10) allowed establishing that with the increase in the thickness of the aluminum layer from 10 to 80 nm, the apparent activation energy E_a_ decreased from 137 ± 3 to 117 ± 2 kJ/mol, and the decimal logarithm of the pre-exponential factor log(A) did so from 14 ± 3 to 11 ± 3 s^−1^ (Table 5).

**Figure 11:**
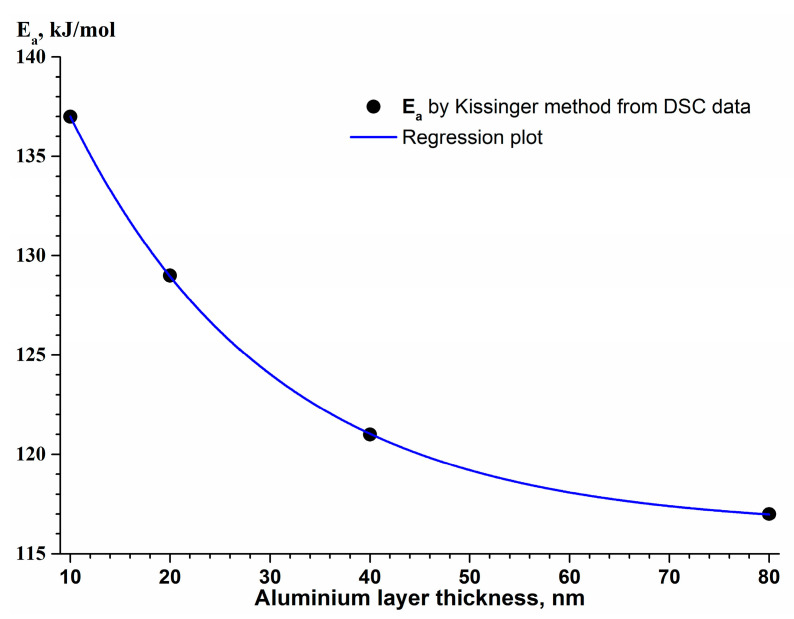
The dependence of the activation energy E_a_ of a-Si crystallization on the aluminum layer thickness in the (Al/Si)_n_ multilayered films.

The analysis of the kinetic parameters (see Table 5) obtained using the Kissinger method shows that there is a relationship between the apparent activation energy E_a_ of silicon crystallization in the (Al/Si)_n_ multilayered films (Figure 11) which can be described by the following equation:

[E_a_] = 116.33 + 33.9 × exp [−0.0493 × l_Al_]

where E_a_ is the apparent activation energy, kJ/mol;

l_Al_ is the thickness of the aluminum layer in the (Al/a-Si)_n_ multilayered sample, nm.

The increase in the activation energy of a-Si crystallization with the decrease in the aluminum layer thickness in the (Al/a-Si)_n_ system is likely to be due to the high degree of defects of the Al thin layers, which is confirmed by the XRD data analysis (see Table 2). The higher degree of Al defects created considerable diffusion restrictions for a-Si atoms, which diffused into the layer of polycrystalline aluminum along the grain boundaries though the interface <Al/a-Si>, resulting in the increase in E_a_.

The obtained values of the apparent activation energy for the silicon crystallization E_a_ = 117–137 kJ/mol (see Table 5) are between 76 kJ/mol, the activation energy of diffusion of Si atoms in the thin Al film [24], and 132 kJ/mol, the activation energy of diffusion of Si atoms along the boundaries of aluminum grains [58]. Here, the obtained activation energy values are significantly lower than the activation energies of diffusion of Al into Si (E_a(Al→Si)_ = 255–335 kJ/mol [1]) and activation energies of silicon self-diffusion (E_a(Si→Si)_ = 396–444 kJ/mol [24]). Thus, the activation energies of silicon crystallization in the (Al/Si)_n_ multilayered thin films obtained in this study indicate that the rate-limiting step of a-Si crystallization is the diffusion of silicon atoms along the boundaries of aluminum grains.

#### 4.2.2. Friedman Analysis

In addition to the Kissinger method [48], the model-free Friedman method [49] allows estimating the complex kinetics of the observed process taking into account the dependence of E_a_ and log(A) on the rate of conversion α, in particular, determining whether the process has many steps; the method also allows estimating the preliminary values of E_a_ and log(A) for individual reaction steps as well as determining the presence of the diffusion control.

The Friedman method is based on the following equation:

$$\ln\lfloor \beta_{i}{\left(\frac{d\alpha }{\mathrm{dT}}\right)}_{\alpha ,\;i}\rfloor =\;\ln\left(f\left(\alpha \right)A_{\alpha }\right)-\frac{E_{\alpha }}{{\mathrm{RT}}_{\alpha ,i}},$$

where T_α,i_ is the temperature corresponding to the rate of conversion α = 0…1 for the i-th measurement;

β_i_ = dT/dt is the linear heating rate for the i-th measurement;

E_α_ is the activation energy at the rate of conversion α;

A_α_ is the pre-exponential factor in the Arrhenius equation at the rate of conversion α;

R is the universal gas constant;

f(α) is the reaction type according to refs. [56,57].

**Figure 12:**
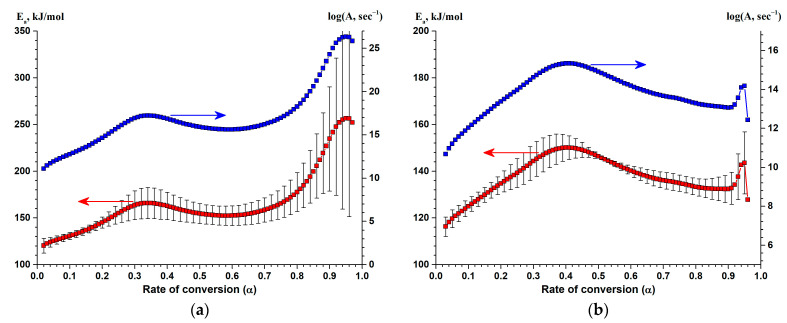

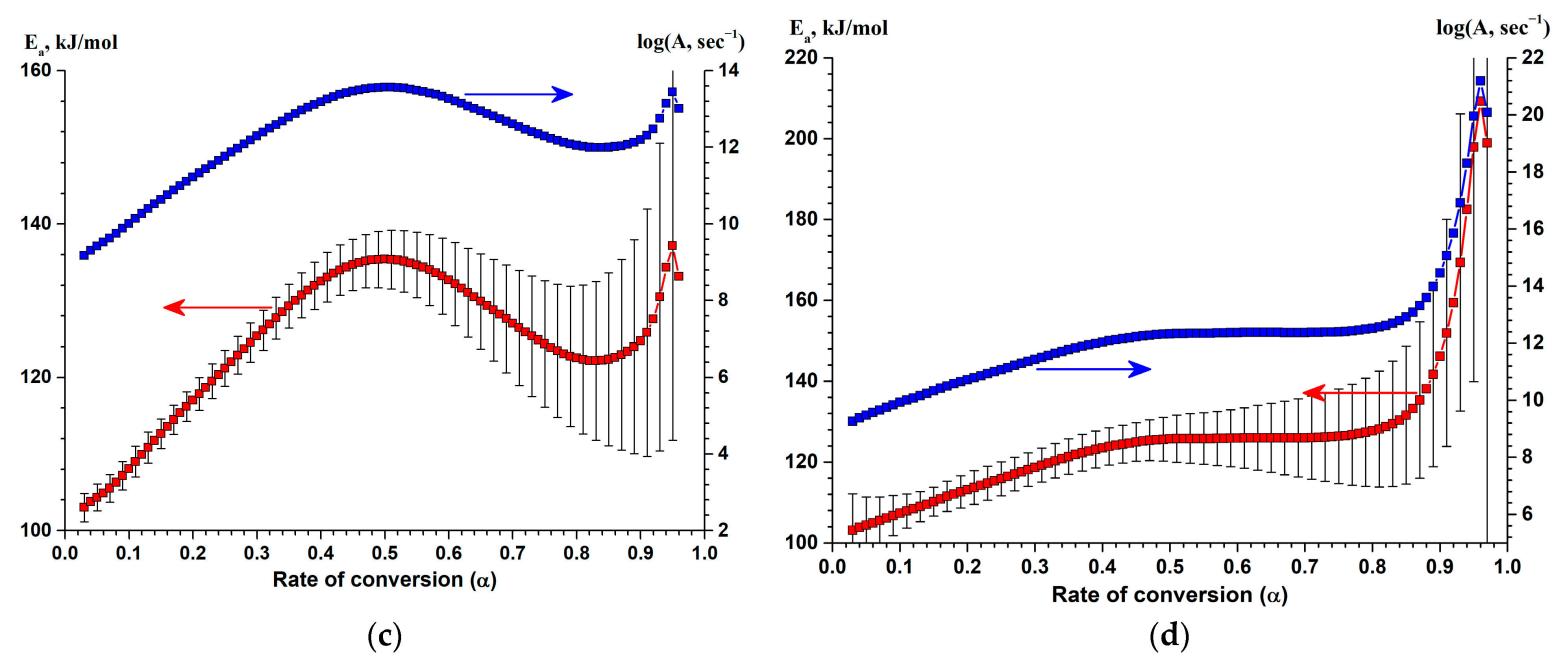
The Friedman analysis of the DSC data for a-Si crystallization in the (Al/a-Si)_n_ multilayered film with the Al layer thickness of 10 nm (**a**), 20 nm (**b**), 40 nm (**c**) and 80 nm (**d**).

The kinetic analysis by means of the model-free Friedman method (Figure 12) shows that the process of silicon crystallization in the (Al/a-Si)_n_ multilayered films is complex: on the curves of the dependence of the apparent activation energy and pre-exponential factor on the rate of conversion, a flat extremum is observed in the region of α = 0.3–0.5. The growth of E_a_ with the increase in the degree at the initial step (α < 0.3) is most likely due to the predominance of the kinetic regime, and the decrease in E_a_ in the region of α = 0.5–0.8 is associated with an increase in the contribution of the diffusion processes. The complex nature of the dependence of E_a_ and log(A) on the rate of conversion is not typical for the purely one-step reaction [55,59], and it can indicate a multi-step process of silicon crystallization.

Moreover, the symbatic nature of the dependences «E_a_–α» and «log(A)–α» allows one to confirm the presence of the kinetic compensation effect [60,61] in the series of solid-state reactions in the Al/a-Si system under study.

### 4.3. Determination of the Most Appropriate Kinetic Model

The kinetic model of the silicon crystallization in the (Al/a-Si)_n_ multilayered films was refined using the multivariate nonlinear regression method [55,56,59,62]. Using the NETZSCH Thermokinetics 3 (v. 2006.08) software package, the kinetic model which best fits the process of silicon crystallization in the (Al/a-Si)_n_ multilayered films was determined, and its kinetic parameters were also calculated (apparent activation energy, pre-exponential factor, reaction order). When choosing the optimal kinetic model, we were guided by the following statistical criteria: the maximum Pearson correlation coefficient R and the Fisher’s test, satisfying the condition F_exp_ < F_crit,0.05_ at a significance level of 0.05. As initial parameters when determining the optimal kinetic parameters, we used the estimation results obtained using the model-free methods of Kissinger and Friedman (see Section 4.2.1 and Section 4.2.2). For the kinetic modeling of individual steps of the solid-phase transformation, use was made of equations of various reaction types [56,63]: «Cn-X» is the nth order reaction with autocatalysis through the reactants, X (X is a product in the complex model); «Bna» is the expanded Prout–Tompkins equation (autocatalytic-type reaction with branching nuclei); «An» is the n-dimensional random nucleation and nucleus/crystallites growth according to the Johnson–Melh–Avrami–Erofeev–Kolmogorov (JMAEK) theory; «Fn» is the n^th^ order reaction; «D3» is three-dimensional diffusion (Jander’s type); «D4» is three-dimensional diffusion (Ginstling–Brounshtein’s type); «R3» is three-dimensional diffusion along grain boundaries.

Since silicon crystallization in the (Al/a-Si)_n_ multilayered thin films is accompanied by the monomodal peak on the DSC curve (Figure 9a–d), regardless of the aluminum layer thickness, preliminary kinetic modeling was performed under the assumption that the process is a single-step one. In this case, the process of silicon crystallization is well described by the equation of the n-th order reaction with autocatalysis “Cn-X” with the apparent activation energies E_a_ = 112.5–131.5 kJ/mol, pre-exponential factors log(A, s^−1^) = 10.34–11.42 and reaction orders *n* = 1.36–2.14 (see Table 6). The kinetic reaction, corresponding to the «Cn-X» type of reaction, is described by the following expression [56]:

$$\frac{\mathrm{de}}{\mathrm{dt}}=-{\mathrm{Ae}}^{n}\left(1+k_{\mathrm{cat}}X\right)\exp\left(-\frac{E_{a}}{\mathrm{RT}}\right)$$

where

t is the time, s;

e = (1 − α) is the starting concentration of the reactant (where α is the rate of conversion);

X = α is the concentration of the intermediate (or final) product;

T is the temperature, K;

k_cat_ is the rate constant of the reaction with autocatalysis by the product X in the complex model;

E_a_ is the apparent activation energy, kJ/mol;

A is the apparent pre-exponential factor, s^−1^;

n is the apparent reaction order;

R is the universal gas constant (8.314642 J·K^−1^·mol^−1^).

However, it should be noted that with an increase in the thickness of the aluminum layer, the discrepancy between the model of one-step silicon crystallization and the experimental data increases (see Table 6), which can indirectly indicate the complication of the crystallization mechanism of a-Si, depending on the thickness of aluminum. In ref. [20], it is shown that silicon crystallization in the Si/Al thin films proceeds in two steps. Taking into account the results of model-free simulation using the Friedman method, the process of silicon crystallization in the (Al/a-Si)_n_ multilayered thin films can also be assumed in this study to occur in several steps.

To study in detail the mechanism of silicon crystallization in the (Al/a-Si)_n_ multilayered thin films, kinetic modeling was carried out in a two-step version. A two-step version of the kinetic modeling of a-Si crystallization was performed with both consecutive and concurrent routes of solid-phase transformation steps (see Table 6). The analysis of the Pearson’s criterion (R) and F-test shows that the transition from the one-step model of silicon crystallization to the two-step one (consecutive or concurrent) is statistically significant (F_exp_ for the two-step process is significantly lower than in the case of the one-step process).

**Figure 13:**
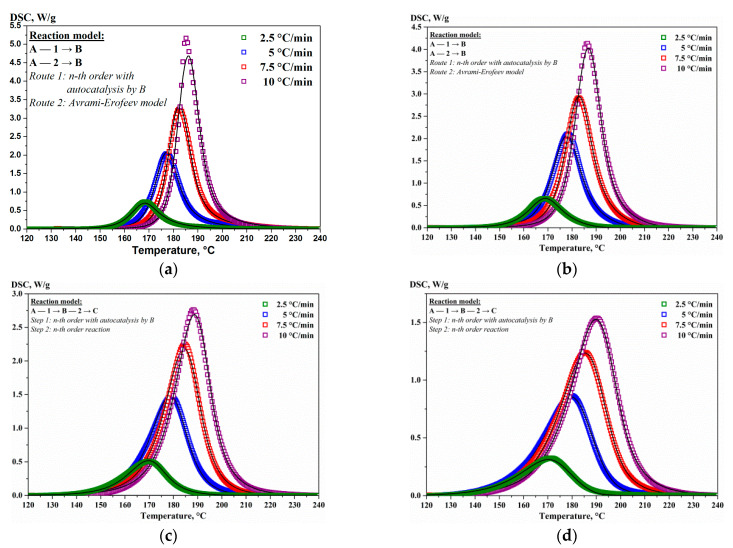
The kinetic modeling (dotted lines) of the DSC measurements (solid lines) for a-Si crystallization in the (Al/a-Si)_n_ multilayered films with the Al thickness of 10 (**a**) and 20 (**b**) (concurrent reactions “Cn-x” + “An”); 40 (**c**) and 80 nm (**d**) (consecutive reactions “Cn-x”→”Fn”).

It is known that the kinetics of nucleation and crystallite growth in massive amorphous materials is described within the framework of the JMAEK theory [64]. However, in the case of nano-sized thin films, one can observe significant deviations from the JMAEK theory [65]. The analysis of the results of kinetic modeling of silicon crystallization in the (Al/a-Si)_n_ multilayered films in the one-step and two-step variants suggests that the main type of solid-phase reaction determining the crystallization process should be a n^th^-order reaction with autocatalysis («Cn-X»). The analysis of the results of kinetic modeling of the silicon crystallization allows one to conclude that in the case of films with the aluminum layer thicknesses of 10 and 20 nm, the process of crystallization proceeds in the form of a concurrent routes of the reaction, each route being characterized by different types of reactions: «Cn-X» and «An» (Figure 13a,b). In turn, in the samples with the aluminum thicknesses of 40 and 80 nm, two-step crystallization is described by consecutive reactions «Cn-X»→«Fn» (Figure 13c,d).

In the case of the concurrent two-step reactions of silicon crystallization in the (Al/a-Si)_n_ multilayered thin films with the aluminum layer thickness of 10–20 nm, the optimal kinetic model is the simultaneous occurrence of reactions in two routes, leading to the formation of crystalline silicon (c-Si):

*Reaction route I* corresponds to the «Cn-X» reaction type, and it is characterized by the activation energies E_a_ = 176.4–187.0 kJ/mol, while the rate-depending mechanism of branching nucleation corresponds to the «Cn-X» reaction [56].

*Reaction route II* is described by the «An» reaction type with the activation energies E_a_ = 45.6–73.7 kJ/mol (see Table 6) and Avrami exponent m = 2.07–2.16. The obtained values of *m* for the (Al/Si)_n_ multilayered films with the aluminum layer thickness of 10–20 nm are in good agreement with the literature data for the Avrami exponent, which are obtained in describing the crystallization of a-Si (or a-SiGe) thin films in the framework of the JMAEK theory [21,45,66,67]. According to refs. [45,68], in the case of a site saturated nucleation process, the Avrami exponent is equal to the dimensionality of the growth. Thus, one can conclude that in the present study (m = 2.07–2.16), the process of «An»-type silicon crystallization is most likely to proceed through two-dimensional crystallite growth controlled by the interface in the presence of supersaturated nucleation centers (formed in reaction route I, the «Cn-X» reaction type).

In ref. [69], it is shown that during the crystallization of pure amorphous silicon films, the activation energy of nucleation is approximately two times higher than the activation energy of crystallite growth. In this study, in the case of the (Al/Si)_n_ multilayered thin films with the aluminum layer thickness of 10–20 nm, the activation energy values of «Cn-X»-type silicon crystallization (E_a_ = 176.4–187.0 kJ/mol) are more than 2.5 times higher than those for «An»-type silicon crystallization (Ea = 45.6–73.7 kJ/mol). Thus, it can be concluded that the «Cn-X» type reaction largely determines the nucleation process, while the “An”-type reaction mainly determines the kinetics of the c-Si crystallite growth.

In the case of the consecutive two-step process (consecutive reactions) of silicon crystallization in the (Al/Si)_n_ multilayered films with the aluminum layer thickness of 40–80 nm, the optimal kinetic model is the consecutive occurrence of the reactions in two steps:

Step 1 corresponds to the «Cn-X» reaction type, and it is characterized by lower apparent activation energies (*E_a_* = 97.4–101.7 kJ/mol) than those in the case of the (Al/Si)_n_ films with the aluminum thickness of 10–20 nm. It can be assumed that the lower E_a_ values of the solid-phase reaction of the «Cn-X» type in the (Al/Si)_n_ films with the aluminum layer thickness of 40–80 nm are associated with a lower energy barrier, which is due to the higher concentration of the a-Si crystallization centers and, as follows from the results of XRD data analysis (see Table 2), lower voltages in the initial films.

Step 2 is described by the equation of formal kinetics of the n-th order “Fn” and most likely corresponds to the recrystallization of primary c-Si crystallites into larger ones—the latter process is largely controlled by diffusion along the grain boundaries, which is confirmed by the low values of the apparent activation energy E_a_ = 30.1–46.7 kJ/mol.

The kinetic equations for the «An» and «Fn» reaction types are given below [56,63]:

For the «An» reaction type:

$$\frac{d\alpha }{\mathrm{dt}}=\;m\left(1-\alpha \right){\left[-\ln\left(1-\alpha \right)\right]}^{1-1/m}\mathrm{Aexp}\left(-\frac{E_{a}}{\mathrm{RT}}\right);$$

For the «Fn» reaction type:

$$\frac{d\alpha }{\mathrm{dt}}={\left(1-\alpha \right)}^{n}\mathrm{Aexp}\left(-\frac{E_{a}}{\mathrm{RT}}\right),$$

where

t is the time, s;

α is the rate of conversion;

T is the temperature, K;

E_a_ is the apparent activation energy, kJ/mol;

A is the apparent pre-exponential factor, s^−1^;

n is the apparent reaction order;

m is the Avrami exponent;

R is the universal gas constant (8.314642 J·K^−1^·mol^−1^).

### 4.4. Summary

Thus, based on the above, one can say that in the case of the (Al/Si)_n_ multilayered films with the aluminum thicknesses of 10–20 nm, the process of silicon crystallization occurs in two concurrent routes (“Cn-x” + “An”), while in the case of the aluminum thicknesses of 40–80 nm, silicon crystallization occurs in two consecutive steps (“Cn-x”→”Fn”). It can be assumed that the main factor determining the mechanism of silicon crystallization is the degree of aluminum defects in the initial state. The analysis of the XRD results of the (Al/a-Si)_n_ multilayered films at the initial state (see Table 2) shows that with the decrease in the aluminum layer thickness, the microstrain values increase, indicating an increase in the degree of aluminum defects. Since silicon crystallization occurs at the boundaries of aluminum grains [70], it can be assumed that the increase in the degree of aluminum defects leads to an increase in the contribution of the two-dimensional mode of silicon growth (see Table 6) at the boundaries of aluminum grains, which is confirmed by the increase in macrostresses in the annealed films with the decrease in the aluminum layer thickness (see Table 3).

## 5. Conclusions

We studied the effect of the aluminum layer thickness on the kinetics of crystallization of amorphous silicon in the (Al/a-Si)_n_ multilayered films with the thickness of an individual aluminum layer from 10 to 80 nm and the fixed thickness of an individual layer of amorphous silicon equal to 80 nm. The studies were carried out during the heating of samples in the temperature range from 40 to 300 °C at a heating rate of 2.5–10 °C/min. Based on the XRD and STA data analysis, amorphous silicon was found to be completely transformed into polycrystalline silicon after heating to 300 °C in all the (Al/a-Si)_n_ multilayered films under study (the rate of conversion α = 1). The analysis of the results obtained by simultaneous thermal analysis (DSC + TG) shows that in the case of the (Al/a-Si)_n_ multilayered films with the aluminum thicknesses of 10–20 nm, the process of silicon crystallization proceeds in two concurrent routes (“Cn-x” + “An”), while in the case of the aluminum thicknesses of 40–80 nm, silicon crystallization occurs in two consecutive steps (“Cn-x”→”Fn”). Based on the data obtained by the methods of XRD, transmission electron microscopy and electron diffraction, the change in the mechanism of crystallization of amorphous silicon initiated by aluminum is assumed to be mainly due to the influence of the degree of aluminum defects in the initial state on the kinetics of silicon crystallization. In particular, an increase in the degree of aluminum defects, in the case of thinner layers, leads to an increase in the contribution of the two-dimensional growth regime of silicon at the grain boundaries of aluminum, leading to the appearance of macrostresses in aluminum and silicon as a result of heating.

The analysis of the results obtained by measuring the electrical resistivity (ρ) upon heating the Al (80 nm)/a-Si (80 nm) thin films as well as those of optical microscopy and simultaneous thermal analysis shows that at the initial stage, silicon diffuses into the aluminum layer only in certain areas rather than doing so uniformly over the entire area of the silicon–aluminum interface.

A direct relationship was established between the thickness of the individual aluminum layer in the (Al/a-Si)_n_ multilayered thin films and the enthalpy of silicon crystallization. The increase in the thickness of the aluminum layer from 10 to 80 nm leads to the increase in the crystallization heat from 12.3 to 16.0 kJ/(mol Si).

As a result of kinetic modeling of silicon crystallization in the (Al/a-Si)_n_ films, the kinetic parameters of silicon crystallization were obtained both in the case of the aluminum layers with the thickness of 10–20 nm (E_a_ = 176.4–187.0 kJ/mol; log(A, s^−1^) = 13.6–15.3; n = 2.32–2.38; log(k_cat_) = 4.9–5.5 for the «Cn-X»-type reaction and E_a_ = 45.6–73.7 kJ/mol; log(A, s^−1^) = 2.1–5.8; m = 2.07–2.16 for the «An»-type reaction), and in the case of the aluminum layers with the thickness of 40–80 nm (E_a_ = 97.4–101.7 kJ/mol; log(A, s^−1^) = 8.6–8.8; n = 0.69–0.78; log(k_cat_) = 1.0–1.5 for the «Cn-X»-type reaction and E_a_ = 30.1–46.7 kJ/mol; log(A, s^−1^) = 1.8–3.6; n = 1.18–1.21 for the «Fn»-type reaction). A decrease was observed in the apparent activation energy (according to Kissinger) of aluminum-induced crystallization of silicon with the increasing aluminum thickness.

## Figures and Tables

**Table 1:** The data of elemental analysis of the (Al/a-Si)_n_ multilayered thin films.

	Al 10 nm/Si 80 nm	Al 20 nm/Si 80 nm	Al 40 nm/Si 80 nm	Al 80 nm/Si 80 nm
Al, wt.%	14.3	24.0	36.6	53.5
Si, wt.%	85.7	76.0	63.4	46.5

**Table 2:** The lattice constant of Al (a), average size of the Al crystallites (❬*D*❭), size distribution width of the Al crystallites (δ❬*D*❭), value of microstrains (❬*ε_Al_*❭_111_), and value of macrostresses (σ_Al_) in the (Al/a-Si)_n_ multilayered thin films at the initial state.

Al Layer Thickness, nm	a, Å	❬*D*❭, nm	δ❬*D*❭, nm	❬*ε_Al_*❭_111_	σ_Al_, *^,^** GPa
10	4.0488(9)	11.4	2.5	0.0069	−0.009
20	4.0503(3)	19.3	6.0	0.0038	0.016
40	4.0480(3)	35.6	12.3	0.0024	−0.024
80	4.0485(3)	61.3	21.3	0.0018	−0.015

* Young modulus of aluminum 70 GPa [42]. ** Tensile stresses are denoted by the sign (+), compressive stresses—by (–).

**Table 3:** The lattice constants (a) of Al and Si, angle (*α* of the rhombohedral lattice of Si, and value of microstrains (σ) in the (Al/Si)_n_ multilayered films after heating to 300 °C.

Sample	a_Al_	σ_Al_ *, GPa	a_Si_	*α*_Si_	σ_Si_ **, GPa
(Al-10/Si-80)	4.0699(5)	0.355	5.399(3)	89.62(5)	−0.879
(Al-20/Si-80)	4.0606(5)	0.194	5.408(4)	89.70(6)	−0.619
(Al-40/Si-80)	4.0550(4)	0.097	5.418(2)	89.81(3)	−0.340
(Al-80/Si-80)	4.0514(2)	0.035	5.421(3)	89.90(3)	−0.251

* Young’s modulus of aluminum 70 GPa (see Table 7.3 in ref. [42]). ** Young’s modulus of polycrystalline silicon 160 GPa (see Table 7.7 in ref. [42]).

**Table 4:** The characteristic temperatures of the DSC peak of a-Si crystallization in the thin film samples of the (Al/a-Si)_n_ system.

Sample	Number of Bilayers	Layer Thickness, nm		Heating Rate β, °C/min	Characteristic Temperatures, °C				−ΔH **, kJ/(mol Si)
		Al	Si		T_conversion-1%_ *	T_onset_	T_max_	T_end_	
(Al-10/Si-80)	60	10	80	2.5	152	157	169	180	12.3 ± 0.4
				5.0	160	167	177	188	
				7.5	165	173	182	193	
				10.0	170	179	185	196	
(Al-20/Si-80)	55	20	80	2.5	146	155	169	184	13.3 ± 0.9
				5.0	155	165	178	190	
				7.5	160	171	183	195	
				10.0	163	176	186	199	
(Al-40/Si-80)	40	40	80	2.5	138	148	170	184	15.8 ± 0.8
				5.0	148	160	179	193	
				7.5	151	168	185	198	
				10.0	152	172	188	202	
(Al-80/Si-80)	35	80	80	2.5	133	145	171	187	16.0 ± 1.0
				5.0	139	155	180	196	
				7.5	141	162	186	202	
				10.0	147	168	190	208	

* Temperature corresponding the rate of conversion equal to 1% (1% of the DSC peak area); ** The enthalpy value of silicon crystallization is calculated as the average of the four measurements at different heating rates taking into account the silicon content in the thin film sample according to the EDS data.

**Table 5:** The estimation of the kinetic parameters of amorphous silicon crystallization in the (Al/Si)_n_ multilayered films using the model-free Kissinger method.

Sample	E_a_, kJ/mol	log(A, s^−1^)	*R* ^2^
(Al-10/Si-80)	137 ± 3	14 ± 3	0.9993
(Al-20/Si-80)	129 ± 5	13 ± 3	0.9974
(Al-40/Si-80)	121 ± 4	12 ± 3	0.9975
(Al-80/Si-80)	117 ± 2	11 ± 3	0.9995

**Table 6:** The results of the kinetic modeling of silicon crystallization in the (Al/a-Si)_n_ multilayered films (the thickness of the individual silicon layer in all the cases is 80 nm).

Sample	Kinetic Parameters of the Reaction Models								F-Test	
One-step reaction: A–(1)→B										
Al thickness, nm	One step (Cn-X)							R^2^	F_exp_	F_crit.__(0.05)_
	E_a_, kJ/mol	log(A,s^−1^)	log(k_cat_)	n (reaction order)						
10	131.5	11.42	2.48	2.14				0.9898	2.26	1.09
20	130.6	11.81	1.84	1.88				0.9950	3.85	1.10
40	119.7	10.97	1.18	1.48				0.9972	2.09	1.09
80	112.5	10.34	0.77	1.36				0.9980	1.68	1.08
Concurrent routes of the reaction: A–(I)→B A–(II)→B′										
Al thickness, nm	Reaction route I (Cn-X)				Reaction route II (An)			R^2^	F_exp_	F_crit.__(0.05)_
	E_a1_, kJ/mol	log(A_1_,s^−1^)	log(k_cat1_)	n_1_ (reaction order)	E_a2_, kJ/mol	log(A_2_,s^−1^)	Avrami exponent *m*			
10	176.4	13.59	5.50	2.38	45.6	2.13	2.07	0.9954	1.00	1.09
20	187.0	15.25	4.88	2.32	73.7	5.78	2.16	0.9986	1.00	1.10
40	For the (Al/a-Si)_n_ samples with the aluminum layer thickness of 40 and 80 nm, the obtained kinetic parameters have no physical or chemical meaning in terms of the given kinetic model since E_a_ > 500 kJ/mol, log(A) are the negative values and π > 50.									
80										
Consecutive route of the reaction: A–(1)→B–(2)→C										
Al thickness, nm	Step 1 (Cn-X)				Step 2 (Fn)			R^2^	F_exp_	F_crit.__(0.05)_
	E_a1_, kJ/mol	log(A_1_,s^−1^)	log(k_cat1_)	n_1_ (reaction order)	E_a2_, kJ/mol	log(A_2_,s^−1^)	n_2_ (reaction order)			
10	127.8	10.88	2.56	1.26	67.2	6.99	2.36	0.9926	1.63	1.09
20	127.2	11.43	1.78	1.14	38.1	3.68	3.35	0.9966	1.68	1.10
40	101.7	8.78	1.46	0.78	46.7	3.60	1.18	0.9986	1.00	1.09
80	97.4	8.60	1.03	0.69	30.1	1.78	1.21	0.9988	1.00	1.08

## Data Availability

Data underlying the results presented in this paper are not publicly available at this time but may be made available by the authors upon reasonable request.
